# SIRT3‐Dependent Actions of Berberine: From Molecular Therapies to Toxicity

**DOI:** 10.1002/jbt.71126

**Published:** 2026-10-11

**Authors:** Dareuosh Shackebaei, Seyed Shahram Miraghaee, Faegheh Farhadi, Abolfazl Akbarzadeh, Fatemeh Yarmohammadi

**Affiliations:** ^1^ Medical Biology Research Center, Health Technology Institute Kermanshah University of Medical Sciences Kermanshah Iran; ^2^ Herbal and Traditional Medicines Research Center, Institute of Pharmaceutical Sciences Kerman University of Medical Sciences Kerman Iran; ^3^ Razi Drug Research Center Iran University of Medical Sciences Tehran Iran

**Keywords:** berberine, deacetylase, metabolic disorders, mitochondria, SIRT3, toxicity

## Abstract

Berberine, an alkaloid with broad bioactive properties, exerts its pleiotropic effects by modulating SIRT3, a mitochondrial NAD^+^‐dependent deacetylase. This review examines the dual role of SIRT3 as a target of berberine in the management of various disorders while also addressing its associated toxicological profiles. The antihyperglycemic effects of berberine are mediated through SIRT3 inhibition, which promotes hyperacetylation of complex I subunits, degradation of MPC1, an increased AMP/ATP ratio, AMPK activation, and suppression of PEPCK1, collectively resulting in the inhibition of gluconeogenesis. In contrast, the hypolipidemic, cardioprotective, and hepatoprotective effects of berberine are associated with SIRT3 activation, which facilitating adipose tissue remodeling, enhancing β‐oxidation in NAFLD, promoting lipophagy in diabetic cardiomyopathy, and mitigates doxorubicin‐induced cardiotoxicity. Furthermore, berberine exhibits antitumor duality, exerting contrasting effects in colorectal cancer and hepatoma models, and demonstrates versatile mitochondrial metabolic reprogramming in polycystic ovarian syndrome. Notably, ocular toxicity associated with berberine has been linked to SIRT3 inhibition in photoreceptors cells. Overall, the therapeutic versatility of berberine is fundamentally associated with its context‐dependent modulation of SIRT3, positioning it as a promising mitochondrial epigenetics modulator for conditions such as metabolic syndrome, NAFLD, and DCM. However, its therapeutic application must be carefully balanced against organ‐specific toxicity risks.

## Graphical abstract

This study reveals that berberine's therapeutic versatility in metabolic, cardiovascular, and hepatic disorders stems from its modulation of the mitochondrial protein Sirt3. Understanding this mechanism explains both its broad efficacy and its organ‐specific toxicity, positioning berberine as a promising mitochondrial epigenetic modulator whose clinical application requires careful balancing of benefits against potential risks.

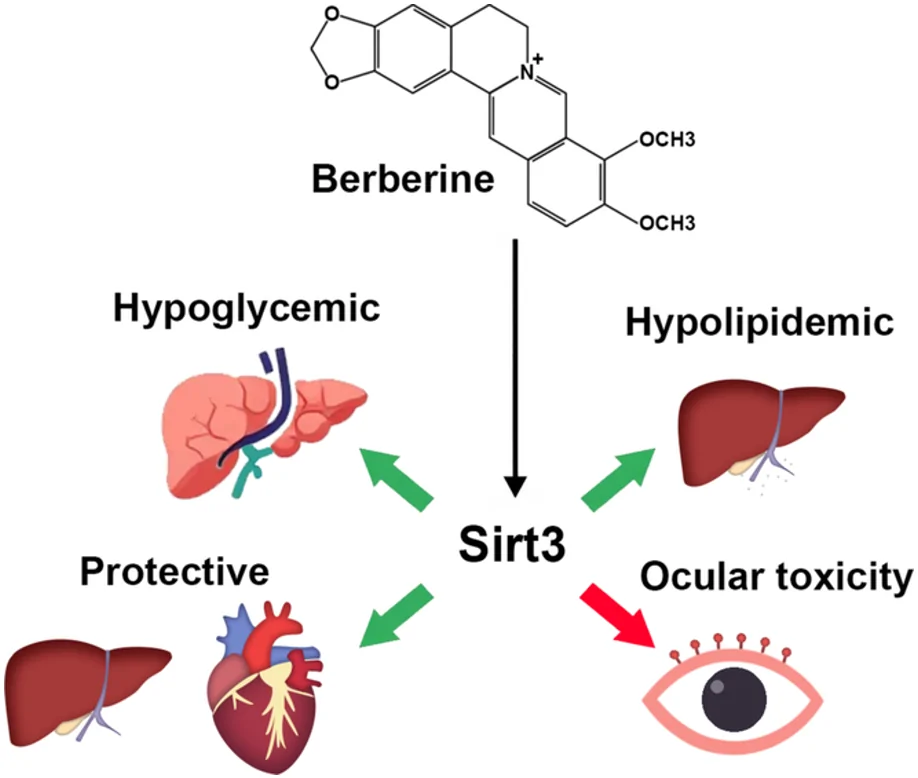

**Abbreviations**
AC: adenylate cyclase
ACC: acetyl‐CoA carboxylase
ALT: alanine aminotransferase
AMPK: AMP‐activated protein kinase
AST: aspartate aminotransferase
Bax: Bcl‐2‐associated X protein
Bim: Bcl‐2 Interacting Mediator of cell death
CAT: catalase
CPT‐1A: carnitine palmitoyltransferase 1 A
CRC: colorectal cancer
CREBBP: cAMP response element‐binding protein binding protein
DCM: diabetic cardiomyopathy
DHT: dihydrotestosterone
ECM: extracellular matrix
ERK: extracellular signal‐regulated kinase
ETC: electron transport chain
eWAT: white adipose tissue
FOXO1: forkhead box protein O1
FOXO3: forkhead box O3
GLUT1: glucose transporter 1
GSR: glutathione reductase
HADCs: histone deacetylases
HDL‐C: high‐density lipoprotein cholesterol
HFD: high‐fat diet
IFN‐γ: interferon gamma
IL: interleukin
iNOS: inducible nitric oxide synthase
KDM6A: lysine demethylase 6 A
LCAD: long‐chain acyl‐CoA dehydrogenase
LD: lipid droplet
LDL‐C: low‐density lipoprotein cholesterol
LKB1: liver kinase B1
LPS: lipopolysaccharide
MAPK: mitogen‐activated protein kinase
MCP‐1: monocyte chemoattractant protein‐1
MEK: mitogen‐activated protein kinase kinase
MEK: mitogen‐activated protein kinase kinase
MFN2: mitofusin‐2
MnSOD: manganese superoxide dismutase
MPC1: mitochondrial pyruvate carrier 1
mtROS: mitochondrial ROS
NAD^+^
: nicotinamide adenine dinucleotide
NAFLD: non‐alcoholic fatty liver disease
NDUFA9: NADH:ubiquinone oxidoreductase subunit A9
NDUFS1: NADH:ubiquinone oxidoreductase core subunit S1
NEFA: non‐esterified fatty acids
NF‐κB: nuclear factor kappa B
NO: nitric oxide
NOD2: nucleotide‐binding oligomerization domain 2
OA: osteoarthritis
OGG1: 8‐oxoguanine DNA glycosylase 1
PCOS: polycystic ovarian syndrome
PEPCK1: phosphoenolpyruvate carboxykinase 1
PGC‐1α: peroxisome proliferator‐activated receptor gamma coactivator 1‐alpha
PINK1: PTEN‐induced kinase 1
PKA: protein kinase A
PP2A: protein phosphatase 2
PP2Cα: protein phosphatase 2 C alpha
PUMA: p53 upregulated modulator of apoptosis
p‐ACC: phosphorylated ACC
p‐AMPK: phosphorylated AMPK
RCP: respiratory control ratio
RIPK2: receptor‐interacting protein kinase 2
ROS: reactive oxygen species
SDHA: succinate dehydrogenase subunit A
SETD7: SET domain‐containing lysine methyltransferase 7
SIRT: sirtuin
SKP2: S‐phase kinase‐associated protein 2
SOD2: superoxide dismutase 2
TC: total cholesterol
TG: triglycerides
TGF‐β1: transforming growth factor‐beta 1
TNF‐α: tumor necrosis factor‐alpha
β‐OX: beta‐oxidation
α‐SMA: alpha smooth muscle actin

## 1 Introduction

Berberine is an isoquinoline alkaloid characterized by a quaternary base structure and chemically denoted as 8‐hydroxy‐1,5,7‐trimethyl‐4‐isoquinolinone [1, 2]. It is predominantly isolated from the Berberidaceae family, including Berberis vulgaris, Berberis aristata, and other Berberis species [3]. The molecule features two benzene rings fused with a tetrahydropyran ring, one of which bears a hydroxyl (‐OH) group, conferring unique physicochemical properties that underpin its diverse biological activities [1] (Figure 1). Berberine exhibits multifaceted pharmacological effects, including potent antioxidant, anti‐inflammatory, antidiabetic, anti‐obesity, anticancer, antimicrobial, cardioprotective, neuroprotective, hepatoprotective, and nephroprotective properties [1, 2, 4, 5, 6, 7, 8, 9]. Despite these diverse pharmacological properties, berberine exhibits poor solubility and limited oral bioavailability [10, 11]. Following absorption, it rapidly distributes to multiple tissues, including the liver, kidneys, heart, lungs, brain, pancreas, muscle, and adipose tissue, with higher concentrations persisting in the liver, heart, brain, muscle, and pancreas than in plasma [5]. Berberine metabolism occurs predominantly through phase I reactions (demethylation), followed by phase II conjugation reactions (glucuronidation, sulfation, and methylation) [10]. The primary phase I metabolites include berberrubine (M1), demethyleneberberine (M2), and jatrorrhizine (M3). Major phase II metabolites include jatrorrhizine glucuronide (M4), jatrorrhizine sulfate (M5), thalifendine glucuronide (M6), berberrubine glucuronide (M7), demethyleneberberine sulfate (M8), and demethyleneberberine glucuronide (M9) [12]. Excretion occurs primarily through feces and to a lesser extent, through bile and urine [10].

**Figure 1:**
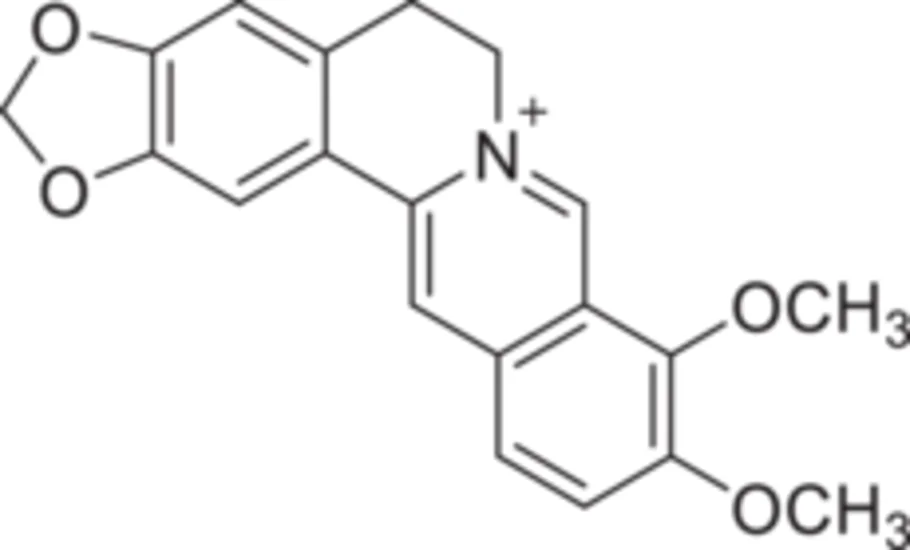
Berberine structure.

Histone deacetylases (HDACs) constitute a superfamily of enzymes that catalyze the removal of acetyl groups from lysine residues on histones and non‐histone proteins, thereby modulating chromatin architecture, transcriptional regulation, and diverse cellular processes [13]. To date, eighteen HDAC isoforms have been identified and are classified into four major groups. The sirtuin (SIRT) subfamily, classified as class III HDACs, comprises seven nicotinamide adenine dinucleotide (NAD)^+^‐ dependent enzymes (Sirt1–Sirt7) characterized by a conserved NAD^+^‐binding catalytic core and isoform‐specific subcellular localization: Sirt1 and Sirt2 are primarily localized in the nucleus and cytoplasm, SIRT3–Sirt5 are mainly localized in the mitochondria, whereas Sirt6 and Sirt7 are predominantly localized in the nucleus [13, 14]. SIRTs target both histones and non‐histone proteins to regulate multiple biological processes, including inflammation, oxidative stress, mitochondrial function, immune responses, and cellular metabolism and proliferation [13, 14].

SIRT3, the primary mitochondrial deacetylase, exerts pivotal control over the acetylation status and enzymatic activity of numerous mitochondrial proteins [15, 16]. It directly deacetylates succinate dehydrogenase subunit A (SDHA) within complex II, as well as components of complexes I and III, thereby enhancing mitochondrial oxidative phosphorylation [13, 15]. In addition, SIRT3 activates forkhead box O3 (FOXO3) to upregulate ROS‐scavenging enzymes such as manganese superoxide dismutase (MnSOD) and catalase (CAT) [17]. It also interacts with the peroxisome proliferator‐activated receptor gamma coactivator 1‐alpha (PGC‐1α) to promote antioxidant enzyme expression, maintain mitochondrial integrity, and counteract excessive reactive oxygen species (ROS) accumulation [13]. Furthermore, SIRT3 plays an important role in regulating immune responses by enhancing T‐cell survival and memory formation, partly through engagement of the AMP‐activated protein kinase (AMPK) signaling pathway [13]. Conversely, SIRT3 deficiency promotes pro‐inflammatory signaling and mitochondrial dysfunction [13].

Since SIRT3 is the primary mitochondrial deacetylase orchestrating redox balance and bioenergetic homeostasis, it serves as a critical convergence point for the pleiotropic effects of berberine [18, 19]. However, the existing literature reveals an apparent paradox: berberine appears to upregulate SIRT3 activity in various models of metabolic and cardiovascular injury, while suppressing it in pathways such as hepatic gluconeogenesis [14, 18, 19, 20, 21, 22]. This context‐dependent regulation challenges a simplistic view of berberine's mechanism and necessitates a more nuanced mechanistic framework [14, 18, 19, 20, 21, 22]. While previous reviews have extensively characterized the broad pharmacological profile of berberine or the general landscape of sirtuin signaling, none have systematically dissected the berberine‐SIRT3 axis to distinguish where SIRT3 functions as a validated therapeutic mediator versus a downstream correlate [17, 23]. This review aims to bridge this knowledge gap by providing a SIRT3‐centered synthesis of current evidence, critically evaluating the therapeutic and toxicological implications of berberine‐mediated SIRT3 modulation, and delineating the unresolved translational challenges in this field [Table 1].

**Table 1:** Effects of berberine on SIRT3 across disease models.

Diseases	Model	Berberine (doses & duration)	Key mechanistic Findings	SIRT3 modulation	Ref
Diabetes	Mice	150 mg/kg/day, 7 days	Increased AMP, reduced cAMP, reduced PEPCK1 and gluconeogenesis, activated AMPK, reduced PKA	↓SIRT3 expression	[18]
	Mouse hepatocytes	5 µM, 6 h			
	HEK293T cell	5 µM, 6 h			
Diabetes	Hepatocytes	10 nM/L, 2 h	Improved glucose/lipid metabolism; reduced complex I; reduced NDUFS1 acetylation	↑SIRT3 activity	[19]
Diabetes	Mice	100 mg/kg, orally for twice	Reduced acetyl CoA; increased MPC1 degradation; reduced MPC1; reduced NAD^+^/NADH	↓SIRT3 expression	[20]
	Mouse hepatocytes	10 μM, 30 min			
Arsenic‐induced diabetes	Rats	100 mg/kg/day, orally for 8 days	Reduced ROS; reduced oxidative stress; improved insulin sensitivity	↓SIRT3 expression	[24]
Arsenic‐induced diabetes	Rats	100 mg/kg/day, orally for 8 days	Increased complex II activity; improved glucose homeostasis; reduced ROS	↑SIRT3 expression	[25]
Obesity	Mice	200 mg/kg/day, orally for 2 weeks	Reduced macrophage infiltration; reduced NF‐κB/MAPKs; reduced miR‐155‐5p	↑SIRT3 expression	[22]
Obesity	Rats	100 mg/kg/day, in the drinking water for 4 weeks	Recovery of OXPHOS efficiency, restoration of calcium buffering capacity, and prevention of energetic deficit	↑SIRT3 expression	[26]
Non‐alcoholic fatty liver disease	Mice	300 mg/kg/day, 4 weeks	Improved β‐oxidation; increased LCAD deacetylation	↑SIRT3 expression	[27]
Non‐alcoholic fatty liver disease	Rats	100 mg/kg/day, orally for 16 weeks	Increased p‐AMPK, p‐ACC, CPT‐1A	↑SIRT3 expression	[28]
Non‐alcoholic fatty liver disease	Mice	25–50 mg/kg/day, orally for 4 weeks	Reduced fibrosis and inflammation; increased α‐SMA expression	↑SIRT3 expression	[29]
Diabetic cardiomyopathy	Mice	136.5 mg/kg, orally for 8 weeks	Enhanced lipophagy, reduced lipotoxicity	↑SIRT3 expression	[30]
	H9C2 cells	10 μM, 24 h			
DOX‐induced cardiotoxicity	H9C2 cells	1–10 μM, 72 h	Reduced P53 and Caspase 3/9; increased SOD2	↑SIRT3 expression	[21]
Osteoarthritis	Mice	0.1 mg/mL, 6–12 weeks	Increased p‐AMPKα and LKB1	↑SIRT3 expression	[31]
	Human chondrocytes	5–25 μM, 18 h			
Cancer	Mice	30–200 mg/kg/day, orally 21 days	Reduced RAS/MEK/ERK/c‐MYC; increased SKP2	↑SIRT3 expression	[32]
Cancer	U266 cell line	40–160 μM, 48 h	Increased SIRT3 expression	↑SIRT3 expression	[33]
	KG1‐α cells	11–44 μM, 48 h			
Cancer	HepG2 cells	100 μM, 24 h	Increased ROS; reduced NAD + /NADH; reduced FOXO1/3a, p53, Bim/PUMA	↓SIRT3 expression	[34]
PCOS	KGN cells	4–12 μM, 24 h	Reduced IL‐6/IL‐1β/TNF‐α; reduced ROS/MDA; reduced miR‐186‐5p	↑SIRT3 expression	[35]
PCOS	KGN cells	12.5–50 μM, 24 h	Increased SIRT3 ubiquitination/degradation; increased ROS; reduced complex I	↓SIRT3 expression	[36]
Ocular toxicity	Zebrafish	20–200 µM, 5 days	Increased ROS; reduced MFN2	↓SIRT3 expression	[37]
	HCE‐T cells	31–250, 24 h			
	Ocular Cell	25–50 μM, 6 h			

## 2 Materials and Methods

This review was conducted using a structured literature search to identify relevant studies investigating the relationship between berberine and SIRT3. Electronic databases including PubMed, Scopus, and Web of Science were systematically searched for relevant publications up to December 2025. The search strategy included combinations of the following keywords: “SIRT3” OR “Sirtuin 3” and “berberine” or “oxyberberine.” Only peer‐reviewed articles published in English were considered. Studies were screened according to three mandatory inclusion criteria [1]: evaluation of berberine or oxyberberine [2], assessment of SIRT3 at the mRNA, protein, or activity level, and [3] investigation within a defined disease or pathophysiological context. Original experimental studies were included, whereas reviews, non‐English articles, and studies lacking direct relevance to berberine and SIRT3 were excluded. The final set comprised 18 studies that met all eligibility criteria (Figure 2).

**Figure 2:**
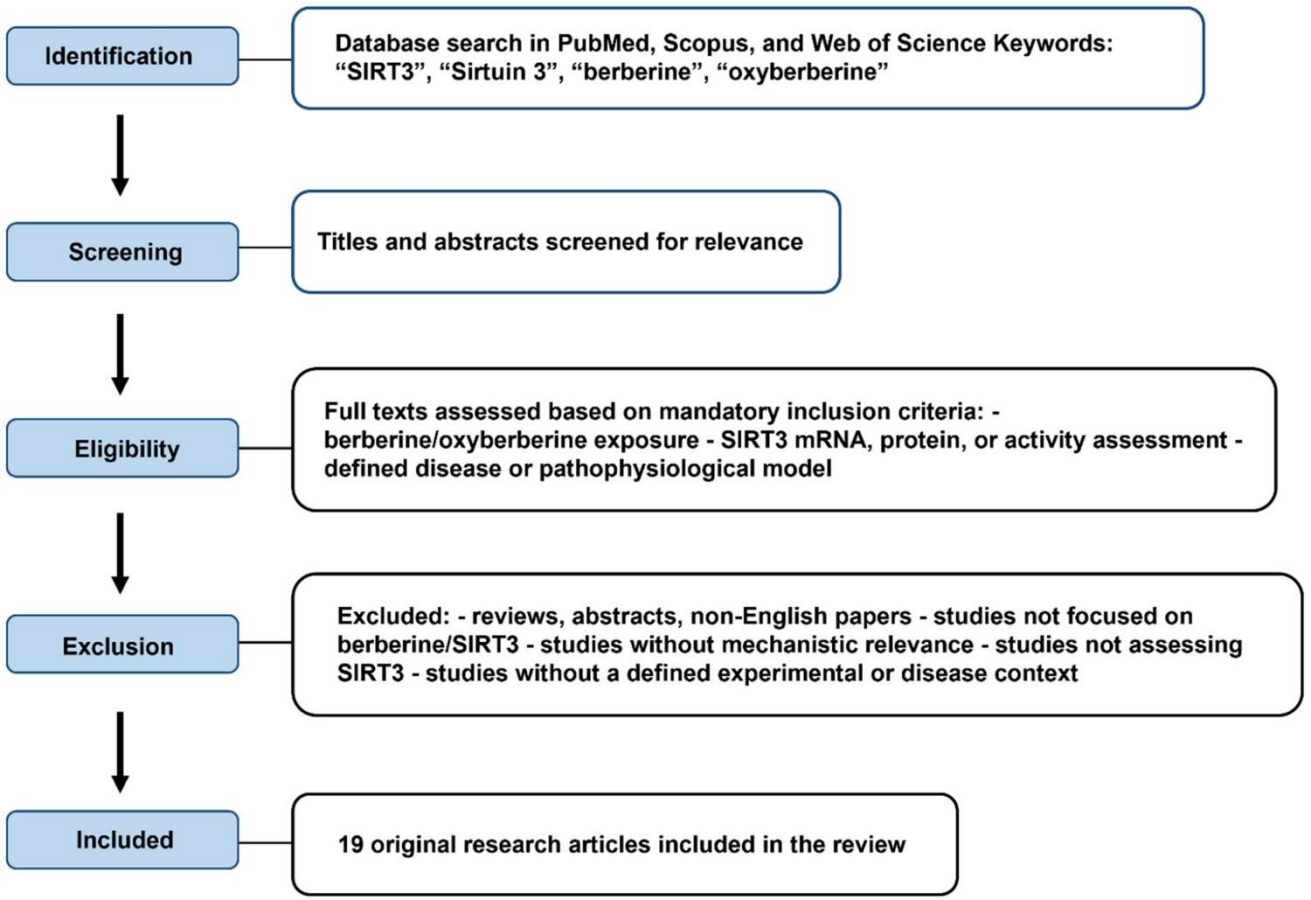
PRISMA flow diagram.

## 3 Therapeutic and Toxicity Effects of Berberine by Targeting Sirt3

### 3.1 Hypoglycemic Effects of Berberine by Targeting SIRT3

Evidence suggests that part of the hypoglycemic effects of berberine, particularly in the liver, is mediated through modulation of SIRT3 and subsequent remodeling of mitochondrial metabolism [18, 19, 20]. Mitochondrial complex I represents the largest multi‐subunit assembly within the electron transport chain (ETC) [38]. This complex consists of 14 core subunits categorized into hydrophilic and hydrophobic polypeptides [38]. The NADH: ubiquinone oxidoreductase subunit A9 (NDUFA9) and NADH: ubiquinone oxidoreductase core subunit S1 (NDUFS1) play critical roles in the assembly or maintenance of complex I stability [19, 39]. SIRT3 acts as a critical regulator of oxidative phosphorylation; by deacetylating key complex I subunits NDUFA9 and NDUFS1, it promotes complex assembly and enhances electron transfer efficiency [19, 40]. Thus, SIRT3 functions as a major cellular energy sensor that translates elevated NAD^+^ levels into optimized ATP production [19, 40]. Berberine has been shown to counteract this mechanism by suppressing SIRT3 activity, resulting in hyperacetylation and impaired function of complex I [18, 19]. This mitochondrial dysfunction subsequently increases the AMP:ATP ratio [18]. Elevated AMP allosterically activates AMPK [18]. Activated AMPK stimulates the translocation of glucose transporters (e.g., GLUT1) to the plasma membrane, thereby enhancing cellular glucose uptake and glycolysis and compensating for mitochondrial energetic deficits [18] (Figure 3a). Therefore, part of the hypoglycemic effect of berberine may be attributed to increased glucose consumption secondary to targeted disruption of mitochondrial metabolism and activation of AMPK [18].

**Figure 3:**
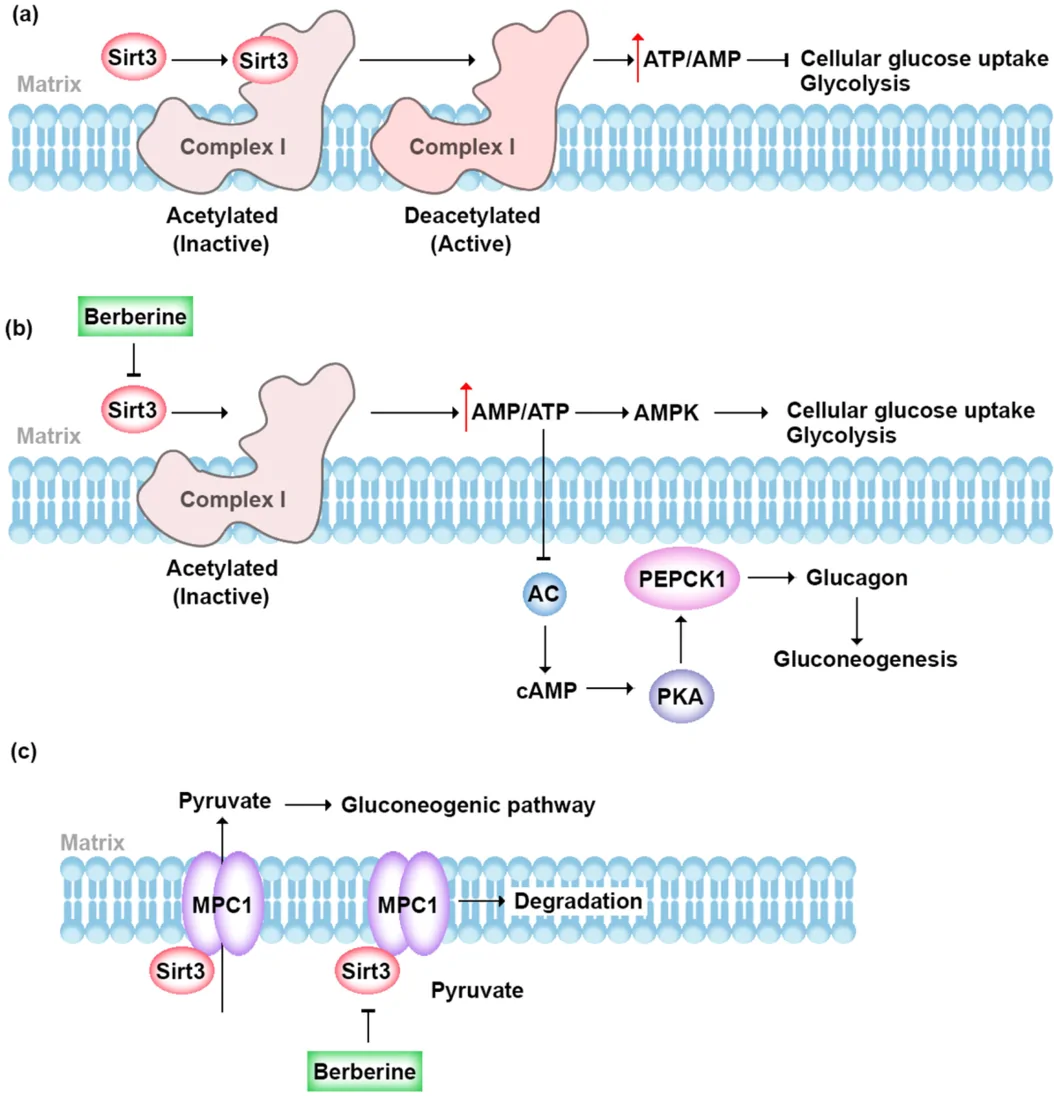
Hypoglycemic effects of berberine by targeting Sirt3, (a) Baseline SIRT3 activity deacetylates complex I subunits, enhancing oxidative phosphorylation and maintaining a high ATP/AMP ratio, which inhibits glucose uptake and glycolysis, (b) Berberine inhibits SIRT3, causing complex I hyperacetylation and dysfunction, increased AMP, and AMPK activation, which promotes glucose uptake/glycolysis. AMP also inhibits AC/cAMP/PKA signaling, leading to PEPCK1 degradation and suppressed gluconeogenesis, and (c) Berberine‐induced SIRT3 inhibition promotes acetylation and degradation of MPC1, restricting mitochondrial pyruvate import and limiting hepatic gluconeogenic substrate supply. AC, adenylate cyclase; AMP, adenosine monophosphate; AMPK, AMP‐activated protein kinase; ATP, adenosine triphosphate; cAMP, *cyclic adenosine monophosphate*; MPC1, mitochondrial pyruvate carrier 1; PEPCK1, phosphoenolpyruvate carboxykinase 1; PKA, protein kinase A; Sirt3, sirtuin 3.

Moreover, AMP acts as a competitive allosteric inhibitor of adenylate cyclase (AC) [18]. This enzyme generates the second messenger cAMP in response to glucagon signaling [18]. Consequently, AMP reduces ATP availability, decreases cAMP production, and attenuates protein kinase A (PKA) activity [18]. PKA normally functions as an intracellular mediator of glucagon signaling by phosphorylating and stabilizing hepatic gluconeogenic targets such as phosphoenolpyruvate carboxykinase 1 (PEPCK1) [18]. Under normal conditions, glucagon‐activated PKA stabilizes PEPCK1 and promotes its deacetylation [18]. However, when PKA activity declines, PEPCK1 undergoes hyperacetylation [18]. This acetylation acts as a molecular signal for E3 ubiquitin ligases, leading to polyubiquitination and subsequent proteasomal degradation of PEPCK1 [18]. Berberine treatment robustly induced this degradation pathway in wild‐type hepatocytes, whereas this effect was markedly attenuated in SIRT3‐deficient systems [18] (Figure 3b).

Mitochondrial pyruvate carrier 1 (MPC1) constitutes one essential subunit of the heteromeric MPC complex embedded in the inner mitochondrial membrane [20]. This complex facilitates the transport of pyruvate from the cytosol into the mitochondrial matrix, thereby linking glycolysis to oxidative phosphorylation and the tricarboxylic acid cycle [20]. SIRT3 has been shown to bind to and deacetylate MPC1, stabilizing the protein and facilitating mitochondrial pyruvate import [20]. By inhibiting SIRT3, berberine preserves the acetylated state of MPC1, marking it for ubiquitin‐mediated proteasomal degradation [20]. The degradation of MPC1 markedly restricts pyruvate transport into the mitochondrial matrix, thereby limiting the primary substrate supply required for hepatic gluconeogenesis [20] (Figure 3c). In addition to its direct metabolic effects, berberine demonstrated protective effects against arsenic‐induced diabetes [24, 25]. In arsenic‐exposed rat models, berberine ameliorated diabetogenic outcomes primarily through its antioxidant properties and modulation of the mitochondrial SIRT3 pathway [24, 25].

Overall, berberine exerts potent antihyperglycemic effects primarily through SIRT3 inhibition, which induces mitochondrial dysfunction and subsequent AMP accumulation [18, 19]. AMP then functions as a dual regulator: it activates AMPK to promote peripheral glucose uptake while simultaneously suppressing the glucagon‐PKA‐PEPCK1 signaling axis to inhibit hepatic gluconeogenesis [18]. Furthermore, berberine suppresses hepatic glucose production by inhibiting SIRT3, which in turn leads to the degradation of MPC1 [20].

Nevertheless, SIRT3 inhibition should not be viewed as an intrinsically and inherently beneficial mechanism. Given the established role of SIRT3 in maintaining mitochondrial integrity, sustaining ATP production, controlling oxidative stress, and preserving metabolic flexibility, chronic or excessive suppression of SIRT3 may be associated with adverse consequences. Theoretically, sustained SIRT3 inhibition could impair oxidative phosphorylation, increase electron leakage, elevate ROS production, and compromise mitochondrial antioxidant defenses, ultimately limiting cellular adaptability to metabolic stress. However, it remains unclear whether berberine, at therapeutically relevant doses, induces the degree of SIRT3 inhibition required to trigger such adverse effects. The clinical significance of this concern likely depends on variables such as dosage, exposure duration, tissue‐specific context, and the baseline oxidative milieu. Consequently, further long‐term, tissue‐specific studies are essential to delineate the clinical relevance of these potential liabilities.

### 3.2 Hypolipidemic Effects of Berberine by Targeting SIRT3

Obesity is a multifaceted disorder characterized by excessive adipose tissue accumulation [41]. It serves as a central driver of various metabolic pathologies, including type 2 diabetes mellitus and cardiovascular diseases [41]. These pathological complications arise primarily from two mechanisms: adipocyte hypertrophy and the heightened release of deleterious adipokines and pro‐inflammatory mediators from expanded adipocytes [41]. In obesity, macrophages infiltrate adipose tissue as part of an inflammatory response to environmental stress [42]. They primarily clear apoptotic cells generated by adipocyte hypertrophy [42]. Although this clearance maintains tissue homeostasis under normal conditions, in obesity pro‐inflammatory signals from enlarged adipocytes recruit additional macrophages and polarize them toward the M1 (pro‐inflammatory) phenotype [42]. These M1 macrophages exacerbate chronic inflammation and secrete excessive inflammatory cytokines [42].

Berberine has emerged as a promising therapeutic agent capable of counteracting these metabolic derangements [22, 26]. In a study utilizing a high‐fat diet (HFD)‐induced obese murine model, Li et al. investigated the pharmacological effects of berberine on adipose tissue remodeling [22]. The research demonstrated that berberine administration effectively ameliorated obesity‐associated metabolic dysfunction in HFD‐fed mice [22]. Key metabolic outcomes included a significant reduction in body weight, adipose tissue mass, and hepatic steatosis, independent of changes in caloric intake [22]. Furthermore, berberine treatment ameliorated systemic glucose homeostasis, as evidenced by reduced fasting blood glucose, as well as improved glucose tolerance, and insulin sensitivity [22]. Serum lipid profiles were also markedly improved, with significant decreases in total cholesterol (TC), triglycerides (TG), low‐density lipoprotein cholesterol (LDL‐C), and non‐esterified fatty acids (NEFA), coupled with an elevation in high‐density lipoprotein cholesterol (HDL‐C) [22]. Berberine also mitigated adipose tissue inflammation by suppressing macrophage infiltration into epididymal white adipose tissue (eWAT) and modulating the M1/M2 macrophage ratio toward an anti‐inflammatory phenotype [22]. This anti‐inflammatory effect is characterized by a reduction in adipocyte hypertrophy and a downregulation of pro‐inflammatory cytokines and chemokine expression [22]. Additionally, it ameliorated abnormal extracellular matrix (ECM) synthesis and deposition in eWAT, thereby enhancing ECM flexibility to facilitate healthy adipose tissue expansion [22]. Berberine also reduced serum levels of liver injury markers alanine aminotransferase (ALT) and aspartate aminotransferase (AST), potentially through downregulation of microRNA‐155‐5p (miR‐155‐5p), which is upregulated in inflamed macrophages and contributes to hepatic steatosis and fibrosis [22]. HFD‐induced elevations in serum and eWAT miR‐155‐5p were completely reversed by berberine [22] (Figure 4a). At the cellular level, emerging evidence indicates that these anti‐inflammatory and immunometabolic modifications are structurally linked to SIRT3 signaling [22]. In macrophage models, stable shRNA‐mediated knockdown of SIRT3 or its pharmacological inhibition using the selective inhibitor AGK7 largely abrogates the capacity of berberine to suppress key inflammatory mediators, including nitric oxide (NO), inducible nitric oxide synthase (iNOS), and pro‐inflammatory cytokines such as interleukin‐6 (IL‐6), tumor necrosis factor‐alpha (TNF‐α), and monocyte chemoattractant protein‐1 (MCP‐1) under lipopolysaccharide (LPS) challenge [22]. Furthermore, berberine enhances SIRT3 expression and enzymatic deacetylase activity in adipose tissue, resulting in the deacetylation of MnSOD/SOD2 and downstream suppression of the nuclear factor‐kappa B (NF‐κB) and mitogen‐activated protein kinase (MAPK) inflammatory cascades [22]. While these cell‐specific loss‐of‐function experiments substantiate a direct, causal role for SIRT3 in coordinating macrophage immunometabolic homeostasis, they do not fully account for the systemic metabolic alterations observed in vivo [22].

**Figure 4:**
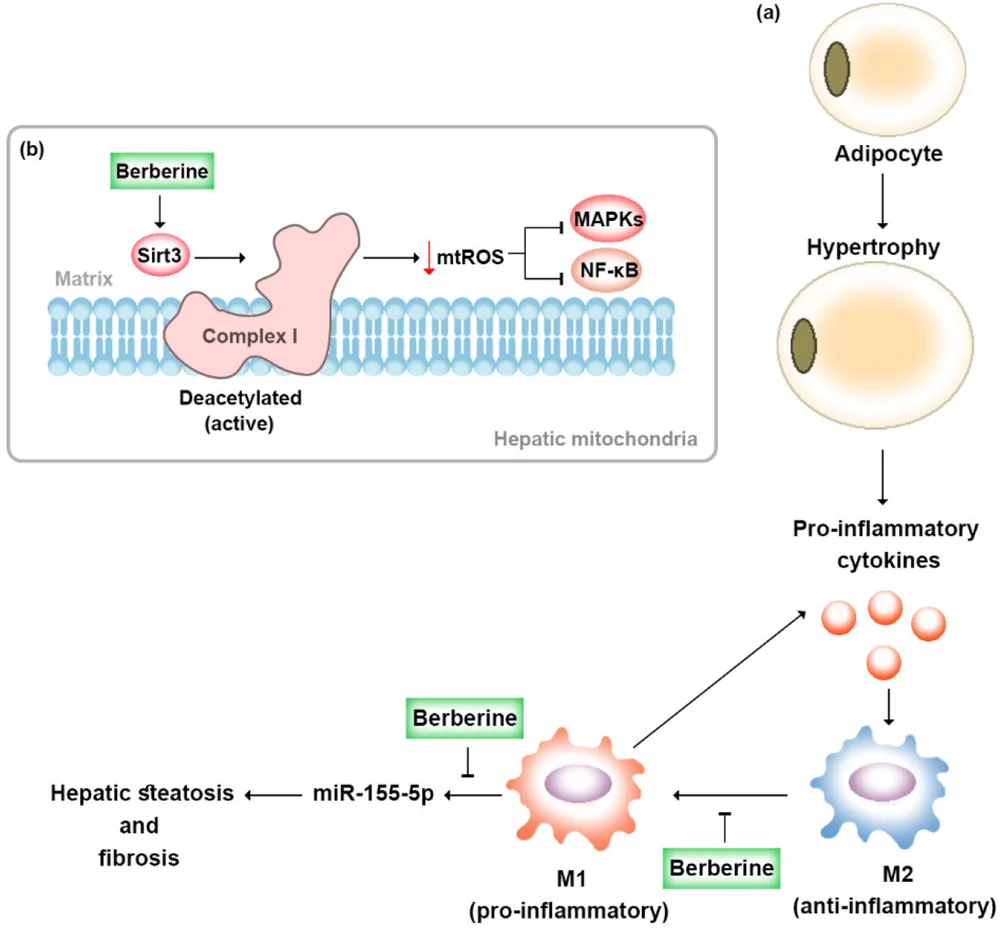
Hypolipidemic effects of berberine by targeting Sirt3, (a) Berberine ameliorates obesity‐induced adipose tissue inflammation by suppressing macrophage infiltration, shifting the M1/M2 macrophage ratio toward an anti‐inflammatory M2 phenotype, and inhibiting miR‐155‐5p, thereby reducing hepatic steatosis and fibrosis, and (b) In hepatic mitochondria, berberine enhances SIRT3 deacetylase activity, leading to MnSOD/SOD2 deacetylation, reduced mtROS production, and subsequent inhibition of the NF‐κB and MAPK inflammatory cascades. These effects restore mitochondrial bioenergetics and likely operate in parallel with other energy sensors such as AMPK to maintain systemic metabolic homeostasis. AMPK, AMP‐activated protein kinase; mtROS, mitochondrial *reactive oxygen species;* NF‐κB, nuclear factor kappa B; Sirt3, sirtuin 3.

Teodoro et al. demonstrated that berberine supplementation in HFD‐fed rats effectively reversed hepatic mitochondrial dysfunction [26]. Berberine restored mitochondrial membrane potential, state 3 respiration, and the respiratory control ratio (RCR) [26]. It also improved mitochondrial calcium retention capacity and mitigated the excessive production of mitochondrial ROS (mtROS) [26]. This bioenergetic improvement correlate with rescued activities of mitochondrial respiratory complexes II, IV, and a restoration of hepatic ATP content [26]. Mechanistically, these functional mitochondrial changes were accompanied by an upregulation of SIRT3 gene expression and a significant rescue of mitochondrial SIRT3 deacetylase activity [26]. Western blot analyses in this study revealed no corresponding increases in total SIRT3 protein levels, suggesting that berberine dynamically enhanced SIRT3 enzymatic activity rather than altering total protein abundance [26]. Importantly, while the macrophage‐specific knockdown studies provide localized causal evidence for SIRT3, the systemic hepatic and mitochondrial improvements documented in whole‐animal models lack direct genetic validation, as no tissue‐specific SIRT3 or AMPK knockouts, knockdowns, or rescue experiments were performed in the rat dietary model [26].

Therefore, while SIRT3 is clearly established as a vital regulatory hub coordinating mitochondrial health and anti‐inflammatory pathways during berberine treatment, its contribution to the overall hypolipidemic phenotype must be interpreted within a broader regulatory network [22, 26]. This network likely operates in parallel with other classical energy sensors, such as AMPK, to orchestrate systemic metabolic homeostasis (Figure 4b).

### 3.3 Hepatoprotective Effects of Berberine by Targeting SIRT3

Non‐alcoholic fatty liver disease (NAFLD) is a progressive hepatic disorder characterized by excessive lipid accumulation in hepatocytes in the absence of excessive alcohol consumption [43]. The disease can progress from simple steatosis to non‐alcoholic steatohepatitis, fibrosis, cirrhosis, and hepatocellular carcinoma [43]. Zhang et al. reported that berberine ameliorates hepatic steatosis in HFD‐fed rats, an effect accompanied by increased hepatic SIRT3 expression and activation of the AMPK/acetyl‐CoA carboxylase (ACC) pathway [28]. In their study, HFD feeding induced obesity, hyperlipidemia, and marked hepatic lipid deposition, accompanied by downregulation of SIRT3, phosphorylated AMPK (p‐AMPK), phosphorylated ACC (p‐ACC), and carnitine palmitoyltransferase 1 A (CPT‐1A) in liver tissue [28]. Mechanistically, SIRT3 has been proposed to facilitate AMPK signaling through its effects on mitochondrial metabolism and cellular NAD^+^/energy homeostasis, which may promote AMPK phosphorylation and downstream ACC inhibition [28]. Phosphorylation of ACC reduces malonyl‐CoA production, thereby relieving inhibition of CPT‐1A, the rate‐limiting enzyme for mitochondrial fatty acid beta‐oxidation (β‐OX) [28]. Collectively, these observations are consistent with the possibility that a SIRT3/AMPK/ACC/CPT‐1‐related pathway contributes to enhanced fatty acid oxidation and reduced lipid accumulation in NAFLD [28]. Berberine treatment reversed these HFD‐induced alterations, including reduced SIRT3 expression and decreased phosphorylation of AMPK and ACC, changes that were accompanied by increased CPT‐1A expression and reduced hepatic lipid accumulation [28]. Consequently, berberine induced fatty acid oxidation and reduced hepatic triglyceride and cholesterol accumulation [28]. Complementing these association‐based findings, Xu et al. provided stronger mechanistic support for SIRT3 involvement in berberine‐induced mitochondrial β‐OX in HFD‐fed mice [27]. They demonstrated that HFD feeding reduced levels of short‐ and medium‐chain acylcarnitines, which are intermediates of fatty acid oxidation [27]. Berberine treatment upregulated SIRT3 expression and decreased the acetylation of its substrate, long‐chain acyl‐CoA dehydrogenase (LCAD), a rate‐limiting enzyme in mitochondrial β‐OX [27]. Hyperacetylation of LCAD under HFD conditions impaired its enzymatic activity, thereby contributing to lipid accumulation [27]. Importantly, the ability of berberine to promote β‐OX was markedly attenuated in SIRT3 knockout mice, providing genetic evidence that SIRT3 is a key mediator of these effects [27].

Overall, available studies suggest that berberine improves hepatic lipid metabolism through mechanisms associated with SIRT3 upregulation, enhanced mitochondrial β‐OX, and modulation of AMPK‐related signaling [27, 28]. However, the strength of evidence is not uniform across studies: while some reports are primarily correlative, genetic data from SIRT3‐deficient models provide more direct support for a causal role of SIRT3 in specific aspects of berberine‐mediated metabolic regulation [27, 28]. It further enhances mitochondrial fatty acid import by inducing ACC phosphorylation [28]. Collectively, these interconnected mechanisms support a multilevel therapeutic action of berberine against hepatic steatosis and NAFLD [27, 28]. Zhao et al. demonstrated that oxyberberine ameliorates carbon tetrachloride‐induced liver fibrosis in mice through SIRT3‐dependent suppression of hepatic inflammation [29]. In vivo, oxyberberine attenuated liver injury and fibrosis, as evidenced by reduced expression of alpha‐smooth muscle actin (α‐SMA) and fibronectin [29]. It also downregulated mRNA and protein levels of pro‐inflammatory cytokines, including IL‐1β, IL‐6, TNF‐α, and interferon gamma (IFN‐γ) [29]. Carbon tetrachloride exposure decreased SIRT3 expression at both transcriptional and translational levels, an effect reversed by oxyberberine [29]. The antifibrotic and anti‐inflammatory effects of oxyberberine were absent in SIRT3 knockout (KO) mice, confirming SIRT3 dependence [29]. In vitro experiments revealed that oxyberberine inhibited cell proliferation and α‐SMA expression in transforming growth factor‐beta 1 (TGF‐β1)‐activated JS‐1 hepatic stellate cells, a mouse cell line commonly employed to study liver fibrosis [29]. Moreover, oxyberberine increased SIRT3 expression, whereas SIRT3 knockdown abolished its inhibitory effects on IL‐1β and IL‐6 secretion in these cells [29]. Together, these findings support the conclusion that oxyberberine mitigates liver fibrosis, at least in part, through SIRT3‐dependent anti‐inflammatory mechanisms [29] (Figure 5a).

**Figure 5:**
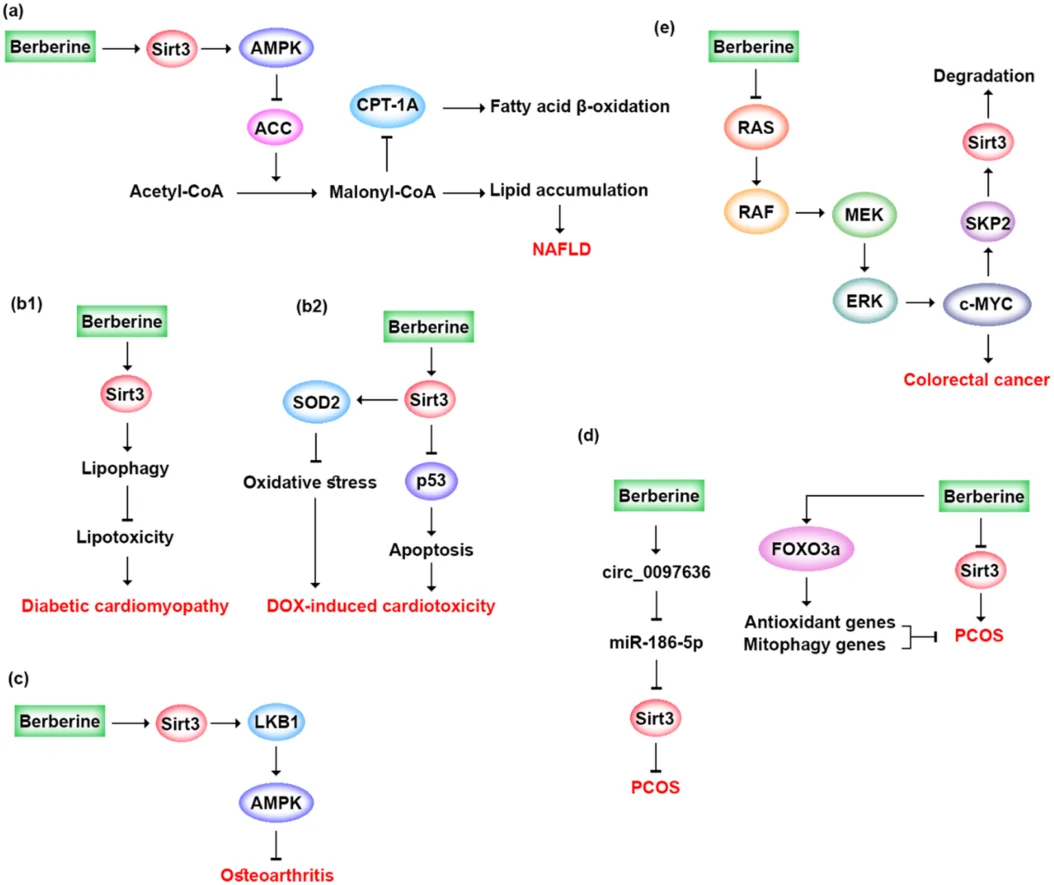
Berberine exerts hepatoprotective, cardioprotective, anti‐osteoarthritic, anticancer, and PCOS‐modulating effects via Sirt3 targeting, (a) Berberine activates SIRT3 to promote AMPK/ACC/CPT‐1A‐mediated fatty acid β‐oxidation, reducing NAFLD and liver fibrosis, (b1) In diabetic cardiomyopathy, SIRT3 upregulation enhances lipophagy to alleviate lipotoxicity, (b2) In doxorubicin cardiotoxicity, SIRT3/SOD2 activation suppresses oxidative stress and p53‐mediated apoptosis, (c) Berberine activates the LKB1/AMPK axis, restoring SIRT1/SIRT3‐associated mitochondrial homeostasis to combat osteoarthritis, (d) In PCOS, berberine context‐dependently either upregulates SIRT3 (via circ_0097636/miR‐186‐5p) for cytoprotection against hyperandrogenism, or induces its transient degradation to trigger AMPK/PINK1‐Parkin‐mediated mitophagy and restore insulin sensitivity, and (e) In colorectal cancer, berberine modulates the RAS/MEK/ERK/c‐MYC/SKP2 axis to increase SIRT3, while in hepatoma cells, SIRT3 suppression promotes apoptosis, highlighting context‐dependent anticancer mechanisms. ACC, acetyl‐CoA carboxylase; AMPK, AMP‐activated protein kinase; CPT‐1A, carnitine palmitoyltransferase 1 A; ERK, extracellular signal‐regulated kinase; FOXO3a, forkhead box O3; LKB1, liver kinase B1; MEK, mitogen‐activated protein kinase kinase; NAFLD, non‐alcoholic fatty liver disease; PCOS, polycystic ovarian syndrome; SKP2, S‐phase kinase‐associated protein 2; Sirt3, sirtuin 3; SOD2, superoxide dismutase 2.

### 3.4 Cardioprotective Effects of Berberine by Targeting Sirt3

Emerging evidence indicates that excessive accumulation of lipid droplets (LDs) and the resulting lipotoxicity are key contributors to the pathogenesis of diabetic cardiomyopathy (DCM) [44]. Lipotoxicity refers to the detrimental effects of excess lipids and their metabolites that accumulate in non‐adipose tissues such as the heart, where lipid uptake or synthesis exceeds the capacity for mitochondrial oxidation and lipid storage [44]. This imbalance leads to LD overload, disruption of cellular signaling, endoplasmic reticulum stress, mitochondrial dysfunction, oxidative stress, inflammation, and ultimately cardiomyocyte dysfunction and death [44]. Lipophagy, a selective form of autophagy in which LDs are sequestered by autophagosomes and degraded within lysosomes, plays a crucial role in maintaining LD homeostasis and preventing lipotoxic injury [44]. Experimental evidence indicates that lipophagy is impaired in DCM, thereby exacerbating cardiac lipotoxicity [30]. SIRT3 has been identified as a key regulator of lipophagy and lipid metabolism in cardiomyocytes, and its expression is downregulated under diabetic conditions [30]. Notably, berberine treatment markedly attenuated diabetes‐induced cardiac dysfunction and hypertrophy in db/db mice in a manner dependent on SIRT3‐mediated lipophagy [30]. These findings suggest that berberine may interrupt a feed‐forward cycle in which impaired lipophagy promotes lipid overload and cardiolipotoxicity, thereby imposing secondary stress on mitochondria. In contrast, restoration whereas restoration of SIRT3 activity enhances lipid clearance and supports mitochondrial homeostasis [30]. Although mitophagy was not directly assessed in this model, the observed effects place SIRT3‐mediated lipophagy within a broader mitochondrial quality‐control framework relevant to cardioprotection in DCM [30] (Figure 5b1).

Doxorubicin is a highly effective anthracycline chemotherapeutic agent widely used to treat a broad spectrum of cancers [45]. However, its clinical utility is significantly limited by dose‐dependent and cumulative cardiotoxicity, which can progress to irreversible congestive heart failure [45]. The pathogenesis of doxorubicin‐induced cardiotoxicity is multifactorial and involves mitochondrial dysfunction, oxidative stress, apoptosis, and dysregulation of autophagy [45, 46]. In line with this, Coelho et al. identified SIRT3 as a major mediator for the protection afforded by berberine in doxorubicin‐treated H9c2 cardiomyoblasts [21]. SIRT3 overexpression preserved mitochondrial network integrity and reduced oxidative stress, while berberine increased SIRT3/Sirt1 levels, enhanced mitochondrial biogenesis markers, modulated autophagy, and inhibited caspase‐9‐ and caspase‐3‐like activation [21]. Berberine upregulates SIRT3, which is linked to suppression of p53‐associated apoptotic signaling and strengthening of antioxidant defense, including activation of SOD2, the principal mitochondrial antioxidant enzyme [9, 21]. Overall, berberine pretreatment restored doxorubicin‐suppressed SIRT3 expression, supporting a SIRT3‐centered mechanism integrating preservation of mitochondrial integrity, attenuation of oxidative stress, enhancement of mitochondrial quality‐control responses, and suppression of apoptosis [21] (Figure 5b2).

Taken together, these findings support the view that SIRT3 functions as a mechanistic nexus linking lipid quality control to mitochondrial resilience in cardiomyocytes [30]. In DCM, SIRT3‐dependent lipophagy may relieve lipid overload and reduce the metabolic pressure imposed on mitochondria, whereas in doxorubicin‐induced injury, SIRT3 directly supports mitochondrial network integrity, antioxidant defense, and survival signaling [21, 30]. Thus, berberine‐mediated activation of SIRT3 appears to protect the heart by coordinately limiting lipotoxic, oxidative, and apoptotic injury [21, 30]. Nevertheless, because mitophagy was not directly demonstrated in either study, it should be discussed as a potential component of the broader mitochondrial quality‐control network rather than as a confirmed mechanism in these models [21, 30].

### 3.5 Anti‐Osteoarthritis Effects of Berberine by Targeting Sirt3

Osteoarthritis (OA) is a degenerative joint disorder characterized by progressive articular cartilage erosion, synovial inflammation, and osteophyte formation [47]. Beyond mechanical stress, OA is increasingly recognized as a metabolically regulated disease in which mitochondrial dysfunction, oxidative imbalance, and inflammatory signaling contribute to chondrocyte injury and cartilage catabolism [47]. In this context, preservation of cellular energy homeostasis and mitochondrial stress responses is critical for maintaining articular cartilage integrity [47].

Berberine has been shown to exert chondroprotective effects in experimental OA, primarily through activation of the liver kinase B1 (LKB1)/AMPK signaling axis [31]. In human primary chondrocytes, berberine induced AMPKα phosphorylation at Thr172, and this effect was abolished by LKB1 siRNA, indicating that LKB1 is required for berberine‐induced AMPK activation [31, 48]. In addition, berberine did not alter the expression of the AMPK‐negative regulatory phosphatases the protein phosphatase 2 (PP2A) or the protein phosphatase 2 C alpha (PP2Cα), supporting a specific upstream effect on the LKB1‐AMPK pathway rather than a nonspecific suppression of AMPK dephosphorylation [31]. Functionally, berberine suppressed IL‐1β‐induced nitric oxide release in chondrocytes, however, this inhibition was only partially lost in AMPKα1‐deficient cells, suggesting that AMPK contributes to, but may not fully account for, the anti‐catabolic action of berberine [31]. Consistently, in destabilization of the medial meniscus (DMM)‐induced post‐traumatic OA, berberine reduced cartilage degradation, synovial hyperplasia, and osteophyte formation in wild‐type mice, and these protective effects were abolished in AMPKα1‐knockout mice [31]. This genetic evidence provides strong support for an essential role of AMPKα1 in mediating the in vivo structural benefits of berberine [31]. At the molecular level, DMM surgery reduced AMPKα phosphorylation and was accompanied by decreased SIRT1 and SIRT3 expression in articular cartilage, whereas berberine reversed these changes in wild‐type mice [31]. In cultured OA chondrocytes, berberine also increased the NAD + /NADH ratio and enhanced the expression of the downstream targets PGC‐1α and 8‐oxoguanine DNA glycosylase 1 (OGG1), consistent with activation of an AMPK‐linked mitochondrial stress‐response program [31]. Although these findings support SIRT3 as part of the downstream signaling network engaged by berberine, the available data do not include SIRT3‐specific loss‐of‐function experiments; therefore, SIRT3 should be interpreted as a candidate downstream mediator rather than a genetically validated essential effector in this study [31]. Collectively, these findings demonstrate that berberine disrupts the self‐perpetuating cascade of energetic stress, inflammatory signaling, and ECM degradation [31]. This therapeutic effect is mediated via the LKB1‐dependent AMPK pathway, which subsequently restores SIRT1/SIRT3‐associated mitochondrial homeostasis [31]. By improving chondrocyte resilience and limiting catabolic responses, berberine may represent a promising strategy for targeting key pathogenic mechanisms in OA [31] (Figure 5c).

### 3.6 Anticancer Effects of Berberine by Targeting SIRT3

Colorectal cancer (CRC) is a leading cause of cancer‑related morbidity and mortality worldwide and is characterized by uncontrolled cell proliferation in the colon or rectum [49, 50]. Its pathogenesis involves genetic mutations, chronic inflammation, dysregulation of key signaling pathways, and alterations in the gut microenvironment, including the intestinal microbiota [49, 50]. c‐MYC, a potent oncoprotein, promotes genomic instability, cell cycle progression, metabolic reprogramming, and sustained tumor proliferation [51]. In this signaling context, RAS functions as an upstream regulator that activates the mitogen‐activated protein kinase kinase (MEK)/extracellular signal‐regulated kinase (ERK) cascade and thereby promotes proliferative and survival signaling [32, 52]. Once activated, ERK enhances oncogenic transcriptional programs in part by phosphorylating c‐MYC, which increases its stability and transcriptional activity [32, 52]. Elevated c‐MYC levels further promote SIRT3 degradation through S‐phase kinase‐associated protein 2 (SKP2)‐dependent mechanisms [32, 52]. Conversely, suppression of ERK destabilizes c‐MYC by disrupting its post‐translational regulation, leading to its downregulation [32, 52]. In a CRC model, the matrine‐berberine combination reduced tumor growth and pathological abnormalities and was associated with decreased RAS and c‐MYC expression and increased SIRT3 expression [32]. The authors therefore proposed that the modulation of the RAS/MEK/ERK–c‐MYC–SIRT3 signaling axis is one of the potential mechanisms underlying the observed antitumor activity [32]. Thus, the concurrent reduction in RAS and c‐MYC and elevation of SIRT3 observed in the CRC model may contribute to the suppression of oncogenic and metabolic signaling [32]. However, because these molecular findings were primarily attributed to the matrine‐berberine combination, they should not be interpreted as definitive evidence that berberine monotherapy produces the same effects [32].

Wang et al. reported that berberine may act as an epigenetic modulator in hematologic cancer cell models, including HL‐60/ADR, KG1‐α and U266 [33]. It altered the expression of several chromatin‐regulatory genes, including SIRT3, the cAMP response element‐binding protein binding protein (CREBBP), EP300, the lysine Demethylase 6 A (KDM6A), and the SET domain‐containing lysine methyltransferase 7 (SETD7), together with changes in histone methylation patterns [33]. These findings suggest that berberine‐associated upregulation of SIRT3 may occur as part of a broader epigenetic response [33]. This study supports the general ability of berberine to modulate SIRT3 expression in selected cancer‐cell contexts [33] (Figure 5e).

In contrast, Shukla et al. demonstrated that berberine suppressed sirtuin expression, including SIRT3, in HepG2 hepatoma cells, thereby disrupting mitochondrial and redox homeostasis [34]. Berberine‐induced sirtuin suppression was associated with excessive ROS generation, reductions in the NAD + /NADH ratio and ATP production, loss of mitochondrial membrane potential, and structural mitochondrial damage [34]. Increased acetylation of forkhead box protein O1 (FOXO1), FOXO3, and p53 also enhanced the expression of the pro‐apoptotic proteins the Bcl‐2 Interacting Mediator of cell death (Bim) and p53 upregulated modulator of apoptosis (PUMA), thereby promoting the Bcl‐2‐associated X protein (Bax) activation and mitochondrial apoptosis [34]. These findings indicate that suppression of sirtuin‐mediated mitochondrial protection may contribute to the antiproliferative and pro‐apoptotic effects of berberine in HepG2 cells [34]. However, because berberine affected multiple sirtuins and the pharmacological rescue experiment involved SRT‐1720, an activator primarily associated with Sirt1, the study does not establish SIRT3 as the sole mediator of berberine‐induced apoptosis [34]. In addition, the relatively high berberine concentration used in this cellular model should be considered when interpreting the translational relevance of these findings [34].

The apparently divergent patterns of SIRT3 regulation observed across these oncological studies are not necessarily contradictory [32, 33, 34]. Rather, they may reflect differences in tumor type, basal SIRT3 expression, metabolic dependencies, mitochondrial function, redox balance, treatment concentration, and experimental regimen [32, 33, 34]. In the CRC model, increased SIRT3 expression accompanied reduced RAS and c‐MYC expression and antitumor activity following combined matrine–berberine treatment [32]. Conversely, in HepG2 hepatoma cells, suppression of sirtuin expression, including SIRT3, impaired mitochondrial bioenergetic and antioxidant defenses, increased oxidative stress, and promoted apoptosis [34]. Adding to this complexity, Wang et al. reported that berberine may act as an epigenetic modulator in hematologic cancer cell lines, including HL‐60/ADR, KG1‐α, and U266 [33]. It altered the expression of several chromatin‐regulatory genes together with changes in histone methylation patterns [33]. Therefore, an increase in SIRT3 may be associated with the attenuation of oncogenic signaling in CRC [32] or occur as part of a broader epigenetic remodeling in hematologic malignancies [33]. In contrast, its suppression may overwhelm mitochondrial stress‐buffering mechanisms and facilitate cancer‐cell death in hepatoma cells [34].

Collectively, these findings indicate that SIRT3 functions as a context‐dependent regulator of tumor metabolism, redox homeostasis, and cell survival rather than as a uniformly tumor‐suppressive or tumor‐promoting factor [32, 33, 34]. Accordingly, the anticancer effects associated with berberine‐related modulation of SIRT3 may arise through distinct mechanisms in different cancer models. The available evidence should therefore be interpreted in light of the specific tumor type and experimental setting. It should not be presented as demonstrating a universal directional effect of berberine on SIRT3 across cancers [32, 33, 34].

### 3.7 Targeting SIRT3 by Berberine in Polycystic Ovarian Syndrome

Polycystic ovarian syndrome (PCOS) is a multifaceted endocrine and metabolic disorder characterized by hyperandrogenism, ovulatory dysfunction, follicular maturation arrest, and insulin resistance, which collectively compromise reproductive success and systemic metabolic homeostasis [53, 54]. At the cellular level, the pathogenesis of PCOS in ovarian follicles is characterized by a vicious cycle wherein chronic inflammation, androgen excess, and lipid accumulation drive mitochondrial dysfunction and oxidative stress, which in turn impair granulosa cell survival and steroidogenic capacity [53, 54]. Recently, SIRT3 has emerged as a key therapeutic target of berberine in the ovarian niche [53, 54]. However, the regulatory effects of berberine on SIRT3 signaling in PCOS models appear highly context‐dependent, exhibiting distinct mechanistic configurations depending on whether the cell is undergoing androgen‐induced oxidative/inflammatory injury or metabolic/insulin‐resistant stress [53, 54].

In the context of hyperandrogenism‐induced follicular injury, simulated by exposing human ovarian granulosa KGN cells to dihydrotestosterone (DHT), berberine exerts cytoprotective, anti‐inflammatory, and anti‐ferroptotic effects through the upregulation of SIRT3 expression [35]. Wang et al. demonstrated that berberine treatment restores the expression of circ_0097636, which was downregulated in the follicular fluid of PCOS patients and in DHT‐treated KGN cells [35]. Mechanistically, using dual‐luciferase reporter and RNA pull‐down assays, the authors established that circ_0097636 functions as a competitive endogenous RNA (ceRNA) that sponges miR‐186‐5p, thereby relieving the post‐transcriptional repression exerted by this microRNA on SIRT3 mRNA [35]. The resulting upregulation of SIRT3 expression and activity counteracts DHT‐induced cellular pathology by restoring mitochondrial membrane potential and metabolic output. Consequently, this axis suppresses intracellular iron accumulation, lipid peroxidation, and reactive oxygen species (ROS) generation, thereby preventing ferroptotic cell death [35]. Furthermore, this SIRT3‐mediated mitochondrial stabilization suppresses downstream inflammatory cascades, as evidenced by marked reductions in the secretion of pro‐inflammatory cytokines, including IL‐6, IL‐1β, and TNF‐α. Concurrently, this mitigation of inflammation promotes granulosa cell proliferation and viability [35]. However, it is important to note that while these findings demonstrate a strong association between the circ_0097636/miR‐186‐5p/SIRT3 pathway and cytoprotection, the study did not perform direct SIRT3 loss‐of‐function (e.g., siRNA/shRNA knockdown or pharmacological inhibition) or genetic rescue experiments in the presence of berberine. Thus, the requirement of SIRT3 activation as an indispensable mediator of berberine's protective effects in this specific model remains a functionally suggestive pathway rather than a genetically validated causal necessity [35].

Conversely, under conditions simulating metabolic dysfunction and insulin resistance in ovarian granulosa cells, berberine appears to engage a distinct and seemingly paradoxical regulatory mechanism involving the downregulation of SIRT3 [36]. Li et al. reported that in insulin‐resistant KGN cells, berberine treatment leads to a dose‐dependent reduction in SIRT3 protein levels [36]. Rather than altering SIRT3 transcription or mRNA stability, berberine accelerates the post‐translational ubiquitination and subsequent proteasomal degradation of SIRT3 [36]. This transient depletion of SIRT3 impairs mitochondrial respiratory complex I activity (without affecting complex II) and induces a controlled depolarization of the mitochondrial inner membrane [36]. The resulting mismatch in the ETC causes a modest, localized elevation in intracellular ROS levels, which acts as a signaling trigger. Concurrently, the reduction in mitochondrial ATP synthesis leads to an accumulation of cellular AMP, which directly activates the AMPK pathway [36]. The activation of AMPK, coupled with the ROS‐mediated activation of the transcription factor FOXO3a, initiates a dual adaptive program [1]: Activated AMPK stimulates glucose transporter translocation, enhancing glucose uptake and restoring insulin sensitivity in the metabolic‐compromised granulosa cells [2]; The transient elevation of ROS and mitochondrial depolarization stabilize PTEN‐induced kinase 1 (PINK1) on the outer mitochondrial membrane, recruiting the E3 ubiquitin ligase Parkin [36]. This activates the PINK1/Parkin‐mediated mitophagy pathway, driving the selective autophagic clearance of damaged, dysfunctional mitochondria. At the same time, the translocation of phosphorylated FOXO3a to the nucleus upregulates essential antioxidant enzymes, including SOD2, CAT, and glutathione reductase (GSR), to prevent runaway oxidative damage [36] (Figure 5d).

Importantly, similar to the findings of Wang et al., the study by Li et al. did not employ genetic ablation (knockout/knockdown) or rescue experiments targeting SIRT3 or AMPK to demonstrate that these specific factors are obligate mediators of the metabolic benefits [35, 36]. Consequently, this metabolic remodeling pathway remains a biochemical association under pharmacological treatment [35, 36].

Rather than representing a direct mechanistic contradiction, these opposing regulatory modes, SIRT3 upregulation for direct cytoprotection versus SIRT3 degradation for metabolic sensitization, reveal a highly sophisticated, context‐dependent thermodynamic tailoring of mitochondrial function by berberine [35]. Within the highly inflamed, androgen‐damaged follicular microenvironment where granulosa cells are actively undergoing apoptosis and ferroptosis, the primary therapeutic requirement is the mitigation of oxidative stress and the preservation of mitochondrial structural integrity [35]. Under these cytopathic conditions, berberine upregulates SIRT3 to deacetylate and activate key mitochondrial enzymes, thereby restoring bioenergetic efficiency and preventing cell death [35]. On the other hand, in metabolic environments burdened by insulin resistance and nutrient overload, where mitochondria can become metabolically rigid, the primary therapeutic need is the restoration of metabolic flexibility [36]. In this context, berberine‐induced transient degradation of SIRT3 functions as a mild mitochondrial stressor [36]. This triggers a transient surge in ROS and AMP levels, which subsequently activates AMPK, facilitates mitophagic clearance of damaged mitochondria, and enhances glucose handling [36].

### 3.8 Potential Ocular Toxicity of Berberine Associated With SIRT3 Modulation

Ocular tissues, including retinal photoreceptors, lens epithelial cells, and corneal epithelial cells, are highly susceptible to xenobiotic injury because of their elevated metabolic demands, dependence on mitochondrial homeostasis, and continuous exposure to environmental stressors [55]. In this setting, berberine appears to exert context‐dependent ocular effects rather than a uniformly toxic or protective profile [55].

Preclinical evidence indicates that berberine can induce ocular toxicity under specific experimental conditions. In human corneal epithelial cells (HCE‐T) and zebrafish, berberine produced marked ocular injury, with photoreceptor damage emerging as a prominent pathological feature [55]. The authors proposed a mechanistic framework involving SIRT3 dysregulation, mitochondrial dysfunction, oxidative stress, impaired autophagy, apoptosis, and inflammation [55]. However, in the absence of direct rescue or loss‐of‐function experiments, SIRT3 should be viewed as a plausible mechanistic contributor rather than a definitively established causal mediator [55].

Translational concern is particularly relevant for topical ocular exposure to berberine‐containing formulations. Available evidence indicates that ophthalmic preparations may contain berberine at concentrations ranging from approximately 0.01%–1.0% (weight/volume), corresponding to roughly 30–3000 micromole, depending on the intended formulation [55]. This issue is further supported by prior work on Goldenseal‐derived alkaloids, in which berberine was reported to cause ultraviolet A‐dependent phototoxicity in human lens epithelial cells [37]. In that study, toxicity was concentration‐dependent, began at low micromolar levels, and was more pronounced in lens epithelial cells than in retinal pigment epithelial cells, indicating a clear tissue‐specific vulnerability [37]. The partial protection observed with antioxidants further suggests that oxidative mechanisms contribute to this phototoxic response [37]. Together, these findings highlight a risk profile that depends on dose, light exposure, tissue type, and route of administration [37].

At the same time, berberine does not appear uniformly deleterious across ocular disease models [56, 57]. In a rat model of lipopolysaccharide‐induced uveitis, oral berberine at 20 and 40 milligram per kilogram reduced inflammatory mediator expression and attenuated leukocyte infiltration without apparent acute toxicity [56]. Likewise, in canine corneal epithelial cells and a model of keratitis, berberine pretreatment at 20 micromole per litre protected against muramyl dipeptide‐induced injury by suppressing the nucleotide‐binding oligomerization domain 2 (NOD2)/the receptor‐interacting protein kinase 2 (RIPK2)/NF‐κB signaling, restoring redox balance, and inhibiting pyroptosis [57]. Notably, neither of these protective studies implicated SIRT3 [56, 57]. Overall, the available evidence supports a context‐dependent ocular profile for berberine. Under phototoxic conditions or in particularly vulnerable tissues such as the lens or photoreceptors, berberine may contribute to mitochondrial and oxidative injury, with SIRT3 implicated as a possible mediator in one toxicity‐oriented study [55]. In contrast, in inflammatory corneal and uveal models, berberine exhibits protective effects through pathways that do not appear to involve SIRT3 [56, 57]. Accordingly, the current literature is more consistent with dose‐, light‐, tissue‐, and route‐dependent effects than with a single uniform safety or toxicity pattern [55, 56, 57]. Further studies are needed to define clinically relevant ocular exposure thresholds, establish human ocular pharmacokinetics, and determine whether SIRT3 plays a causal role in berberine‐associated ocular toxicity across different tissues and exposure conditions (Figure 6).

**Figure 6:**
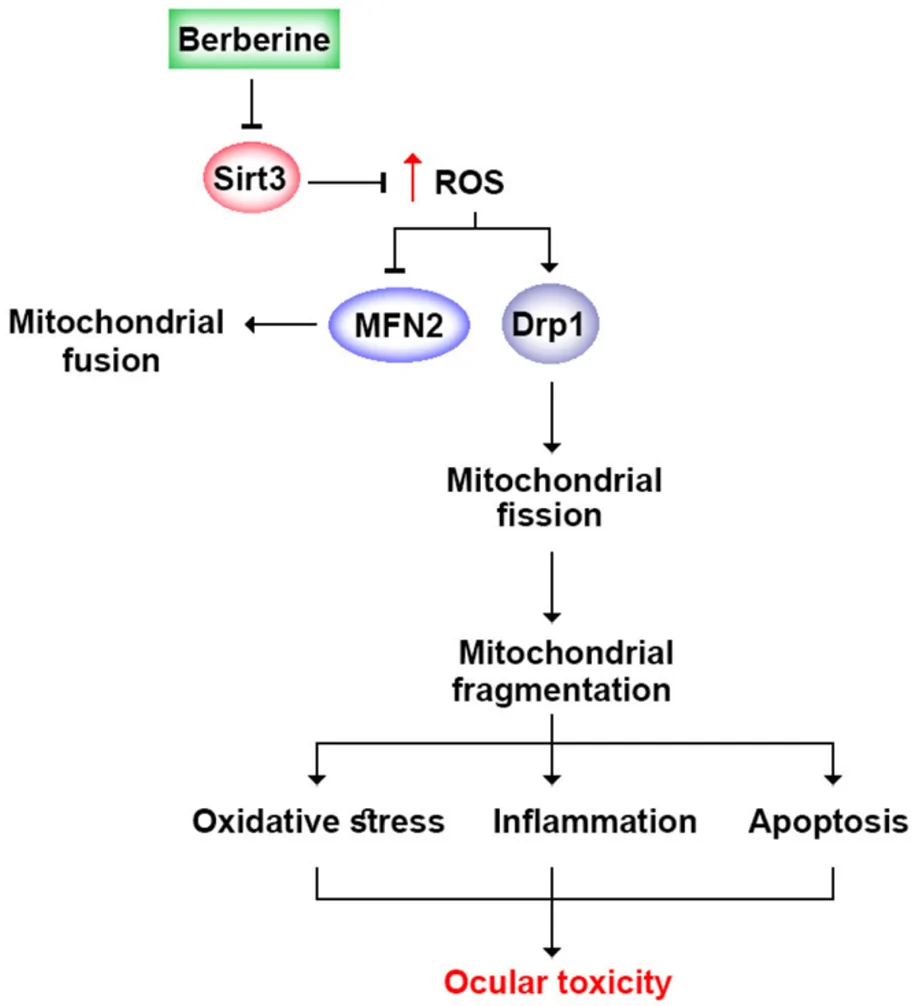
Ocular toxicity of berberine by targeting Sirt3. Drp1, *dynamin‐related protein 1; MFN2, mitofusin‐2;* ROS, *reactive oxygen species;* Sirt3, sirtuin 3.

## 4 Determinants of Context‐Dependent SIRT3 Regulation by Berberine

The contrasting activation and inhibition of SIRT3 by berberine across different experimental models are not contradictory; rather, they reflect a highly orchestrated, context‐dependent response. Based on the current literature, several key biological and experimental factors dictate whether berberine upregulates or downregulates SIRT3.

### 4.1 Tissue Specificity and Basal Metabolic State

Different tissues possess distinct mitochondrial densities, baseline redox thresholds, and metabolic dependencies.

#### 4.1.1 Cytoprotective/Restorative Contexts

In tissues with high mitochondrial reliance and susceptibility to lipotoxicity or oxidative damage (such as cardiomyocytes in DCM or HFD‐fed hepatocytes), the baseline state is characterized by severe mitochondrial stress. Here, berberine upregulates SIRT3 to facilitate adaptive mitochondrial remodeling, activate SOD2, and promote fatty acid oxidation [21, 22, 28, 30].

#### 4.1.2 Reprogramming/Suppressive Contexts

In metabolic programs like active hepatic gluconeogenesis, suppressing mitochondrial pyruvate transport and oxidative ATP output is necessary to reduce glucose output. In these contexts, berberine suppresses SIRT3, facilitating the proteasomal degradation of gluconeogenic enzymes like PEPCK1 and MPC1 [18, 20].

### 4.2 Disease Stage and Redox Environment

The intensity of the cellular redox stress is a major driver of SIRT3 directionality.

#### 4.2.1 Mild‐to‐Moderate Stress

Under compensated metabolic stress, berberine promotes adaptive upstream pathways (such as AMPK activation and increased NAD^+^ availability), which upregulate SIRT3 expression and activity to support mitochondrial repair [28, 31].

#### 4.2.2 Severe/Decompensated Stress or Toxicity

In environments characterized by massive ROS accumulation or structural mitochondrial damage (such as HepG2 hepatoma cells under high‐dose treatment or corneal epithelial cells under phototoxic stress), the severe stress response may trigger ubiquitin‐mediated proteasomal degradation of SIRT3, leading to its depletion and subsequent apoptosis [34, 36].

### 4.3 Biphasic Dose‐Dependency

The pharmacological concentration of berberine dictates the cellular signaling outcome.

#### 4.3.1 Low‐to‐Moderate Concentrations

These typically induce an adaptive, hermetic response. By activating AMPK and restoring NAD^+^/NADH ratios, low doses support mitochondrial quality control and sustain or enhance SIRT3 levels [22, 28, 35].

#### 4.3.2 High Concentrations

High concentrations can cause partial inhibition of mitochondrial respiratory complex I, triggering an acute energy crisis (elevated AMP/ATP ratio) and a transient surge in ROS. In insulin‐resistant granulosa cells, this metabolic overload accelerates the post‐translational degradation of SIRT3, using the resulting transient mitochondrial depolarization to activate downstream PINK1/Parkin‐mediated mitophagy [36].

### 4.4 Upstream Signaling and Post‐Translational Modifiers

Berberine does not appear to bind uniformly to SIRT3 in all tissues; instead, it modulates upstream networks.

#### 4.4.1 Transcriptional/Epigenetic Regulation

Berberine can act as an epigenetic modulator, altering histone methylation and chromatin‐regulatory genes (such as CREBBP and EP300) to upregulate SIRT3 expression [33].

#### 4.4.2 Post‐Translational Fate

The cellular abundance of E3 ubiquitin ligases (e.g., SKP2) and the status of oncogenic drivers (such as c‐MYC and the RAS/MEK/ERK cascade in colorectal cancer) heavily influence whether SIRT3 is stabilized or targeted for proteasomal degradation [32, 36].

## 5 Discussion

The present review suggests that berberine modulates SIRT3 signaling in a highly context‐dependent manner rather than functioning as a uniform activator or inhibitor [18, 22, 28, 30, 32, 34]. Across metabolic, inflammatory, degenerative, and oncologic models, the direction and magnitude of SIRT3 modulation appear to vary according to tissue‐specific metabolic demands, mitochondrial functional state, redox balance, and exposure conditions [22, 28, 30, 32, 34, 35, 36].

In metabolic disorders characterized by mitochondrial dysfunction and oxidative stress (such as obesity, NAFLD, DCM, and osteoarthritis), berberine has frequently been reported to enhance SIRT3 expression or activity [22, 26, 27, 28, 30, 31]. Restoration of SIRT3 signaling in these contexts has been associated with improved mitochondrial β‐oxidation, activation of antioxidant defenses (including SOD2), reduction of mitochondrial ROS, and enhanced mitochondrial quality control [21, 28, 30, 31]. These effects appear to be largely mediated through AMPK activation, improved NAD^+^ availability, and reinforcement of mitochondrial homeostasis [28, 31]. Under such conditions, SIRT3 activation may represent an adaptive response that supports mitochondrial repair and metabolic stabilization [28, 30, 31]. Taken together, these findings suggest that SIRT3 inhibition may contribute to berberine's hypoglycemic actions primarily by suppressing hepatic gluconeogenesis [18, 19, 20], whereas SIRT3 activation under oxidative or lipid overload conditions may facilitate mitochondrial repair and cardiometabolic protection [22, 28, 30]. Thus, the opposing SIRT3 directions reported across studies may reflect distinct metabolic objectives rather than contradictory pharmacological effects [18, 22, 28, 30].

Conversely, in models of active hepatic gluconeogenesis, berberine has been reported to reduce SIRT3 expression or activity [18, 19, 20]. This suppression is associated with decreased PEPCK1 expression, altered NAD^+^/NADH ratios, and inhibition of glucagon‐dependent signaling [18, 19, 20]. Rather than reflecting mitochondrial impairment, this response may represent coordinated metabolic reprogramming that limits oxidative ATP production and reduces hepatic glucose output [17, 18]. Thus, SIRT3 downregulation in this context appears to be mechanistically consistent with berberine's glucose‐lowering action and may be interpreted as a metabolically adaptive response rather than a detrimental effect [18, 19, 20].

Tissue‐specific mitochondrial biology further may contribute to heterogeneity in SIRT3 responses [28, 30, 32, 34]. The liver, heart, ovary, retina, and cancer cells each possess distinct mitochondrial regulatory networks and baseline redox thresholds [28, 30, 32, 34, 35]. In the context of PCOS, berberine has been reported to augment SIRT3‐mediated cytoprotection in ovarian granulosa cells at lower concentrations [35]. Conversely, elevated concentrations have been shown to trigger ubiquitin‐dependent degradation of SIRT3, leading to metabolic sensitization [36]. Similar bidirectional patterns are observed in cancer systems, where SIRT3 may exerts either tumor‐suppressive or oncogenic‐supporting activities in a context‐dependent manner, dictated by mitochondrial bioenergetics and underlying oncogenic drivers [32, 34]. These findings suggest that SIRT3 modulation by berberine is dictated by baseline metabolic state of the cell rather than a predetermined pharmacological trajectory [32, 34, 35, 36].

The structural and regulatory diversity of SIRT3 may provide an additional explanatory layer [58, 59]. SIRT3 exists as a full‐length precursor (~44 kilodalton) containing a mitochondrial targeting sequence and a processed, highly active mitochondrial isoform (~28 kilodalton) [58, 59]. Because the precursor exhibits minimal catalytic activity prior to mitochondrial import, the biological outcome may depend on mitochondrial processing efficiency and isoform balance [58, 59]. Moreover, post‐translational modifications (including AMPK‐dependent phosphorylation, acetylation, ubiquitin‐mediated degradation, succinylation, and malonylation) regulate SIRT3 stability, localization, and substrate specificity [59]. These regulatory mechanisms suggest that functional SIRT3 activity cannot be inferred solely from total expression levels [59]. Instead, isoform composition and post‐translational modification status may help determine whether berberine stabilizes SIRT3 to promote mitochondrial protection or facilitates its turnover to enable metabolic reprogramming [58, 59].

Importantly, current evidence appears to support an indirect regulatory model [18, 28, 31]. No consistent experimental data have yet demonstrated direct binding of berberine to the catalytic pocket of SIRT3 [18, 28, 37]. Instead, berberine orchestrates a range of upstream metabolic processes_specifically AMPK signaling, mitochondrial respiratory flux, redox homeostasis, and the NAD^+^/NADH ratio_which collectively converge to modulate SIRT3 expression and enzymatic activity [28, 31]. Through this indirect metabolic rewiring, berberine may position SIRT3 either as a mitochondrial stabilizer in oxidative stress conditions or as a modulatory node during metabolic adaptation [22, 28, 30].

Interpretation of these findings should also take into account pharmacokinetic constraints [37]. Berberine is characterized by poor oral bioavailability, extensive first‐pass metabolism, and relatively low systemic plasma concentrations [37]. Many studies utilize superaphysiological concentrations of berberine, potentially confounding the interpretation of SIRT3‐mediated responses and mitochondrial outcomes by failing to reflect pharmacological realities in humans [37]. Without pharmacokinetic guidance, it remains difficult to determine which mechanistic pathways are physiologically relevant in humans [37].

In addition, extensive metabolic transformation gives rise to derivatives such as berberrubine, demethyleneberberine, thalifendine, and jatrorrhizine, some of which may reach higher circulating levels than the parent compound [37]. These metabolites may possess distinct mitochondrial and redox‐modulating properties, yet their specific interactions with SIRT3 remain insufficiently characterized [37]. Variability in metabolite formation across tissues and species may contribute to inconsistencies in reported outcomes [37].

Dose‐response relationships may represent another critical determinant [22, 28]. At lower concentrations, berberine appears to induce adaptive AMPK‐dependent responses and support mitochondrial homeostasis [22, 28, 31], frequently accompanied by preserved or enhanced SIRT3 activity [22, 28, 35]. At higher concentrations, stronger mitochondrial stress—including partial respiratory inhibition and increased ROS generation—may alter SIRT3 stability or promote degradation [18, 34, 36, 37]. These biphasic effects highlight the importance of exposure‐dependent interpretation [22, 28, 37].

Despite substantial preclinical progress, significant translational limitations remain [18, 22, 28, 30, 34, 36, 37]. Most evidence derives from cell lines and rodent models, with limited validation in human tissues [18, 22, 28, 30, 34, 36, 37]. In many studies, SIRT3 involvement is inferred from changes in expression rather than confirmed through genetic manipulation [28, 30]. Moreover, few investigations integrate pharmacokinetic measurements or metabolite profiling with mechanistic analyses [37]. Addressing these gaps will require pharmacokinetic‐guided experimental designs, human biomarker studies, and causal validation using SIRT3‐specific perturbation approaches (Figure 7).

**Figure 7:**
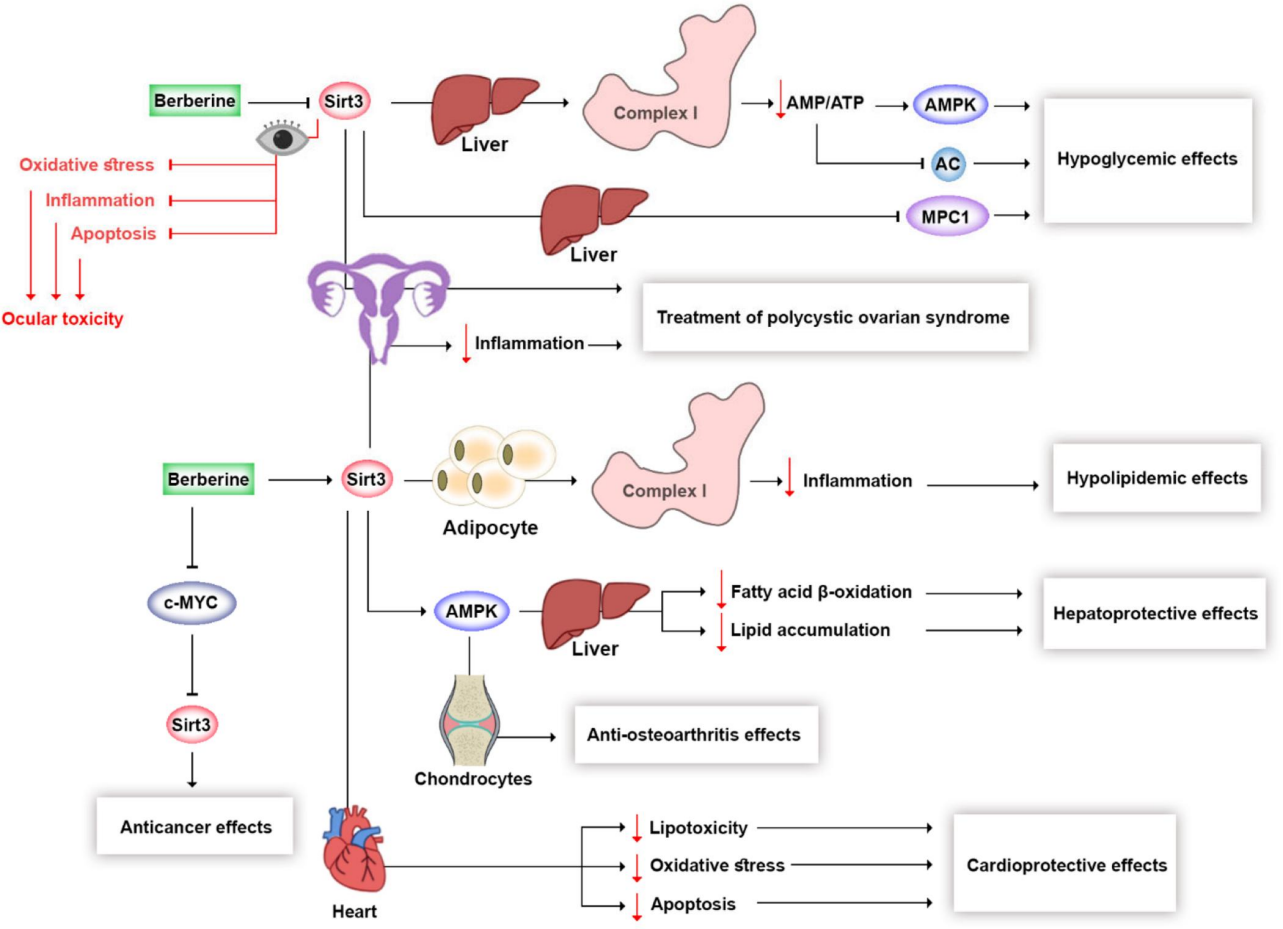
Context‐dependent bidirectional regulation of SIRT3 by berberine and its major phenotypic outcomes. Berberine has been reported to either activate/upregulate or inhibit/downregulate SIRT3 depending on tissue type, disease model, metabolic state, and experimental conditions. In most cytoprotective settings, SIRT3 activation is associated with improved mitochondrial function, enhanced AMPK‐linked metabolic adaptation, reduced lipid accumulation, and attenuation of oxidative stress, inflammation, and apoptosis, contributing to hepatoprotective, cardioprotective, anti‐osteoarthritic, hypolipidemic, and selected anticancer effects. Conversely, in liver models related to suppression of gluconeogenesis and in ocular toxicity‐associated systems, SIRT3 inhibition/downregulation has been linked to anti‐hyperglycemic or toxicological outcomes. PCOS‐associated models show bidirectional patterns, with both SIRT3 activation and downregulation reported under different experimental contexts. Because the underlying mechanisms are not equally established across all systems, this schematic should be interpreted as an integrative conceptual framework rather than a definitive universal pathway map.

## 6 Methodological Quality and Limitations of Mechanistic Evidence

While the context‐dependent determinants discussed in Section 3 suggest how tissue, dose, and metabolic state direct SIRT3 regulation, the literature summarized in this review varies substantially in evidential strength. In several studies, conclusions regarding SIRT3 involvement are based primarily on changes in SIRT3 mRNA or protein levels following berberine treatment, as observed in models of obesity, NAFLD, osteoarthritis, and certain cancer systems [22, 27, 28, 29, 31, 32, 33, 34]. Although such expression shifts suggest a regulatory relationship, they do not demonstrate that SIRT3 is required for the biological effects attributed to berberine [22, 27, 28, 29, 31, 32, 33, 34]. Other studies infer SIRT3 involvement indirectly through downstream markers (e.g., decreased LCAD acetylation, activation of SOD2, or reductions in mtROS) [21, 27, 35]. While these signaling changes are compatible with enhanced SIRT3 activity, they remain associative unless validated by genetic manipulation [21, 27, 35].

Multiple studies, however, provide direct causal support by demonstrating that the pharmacological effects of berberine are diminished or abolished in SIRT3 knockout or knockdown systems [27, 29]. These include the loss of hypoglycemic actions of berberine in SIRT3‑deficient hepatocytes, impaired induction of β‑oxidation in SIRT3‑null mice under NAFLD conditions, reduced cardioprotection in diabetic or doxorubicin‐treated cardiomyocytes lacking SIRT3, the absence of antifibrotic efficacy of oxyberberine in SIRT3‑deficient mice, and the failure of berberine to suppress inflammatory cytokines in TGF‑β1–activated hepatic stellate cells following SIRT3 knockdown [18, 19, 21, 27, 29]. These findings collectively support the premise that SIRT3 is mechanistically necessary for several of the metabolic and anti‐inflammatory effects of berberine [18, 19]. Notably, the forced overexpression of SIRT3 proved sufficient to abrogate berberine‐responsive phenotypes, specifically mitochondrial fragmentation, p53 accumulation, and caspase activation, within doxorubicin‐challenged H9c2 cardiomyoblasts [21].

In ocular toxicity models, competitive docking and enzymatic activity assays indicate that berberine binds to the catalytic pocket of SIRT3 with relatively high affinity, supporting a direct inhibitory mechanism [37]. These biochemical data provide valuable evidence of molecular interaction [37]; however, whether this direct binding is preserved across other tissue types and physiological contexts remains to be confirmed.

Overall, distinguishing these tiers of evidence reveals substantial variability in the mechanistic certainty supporting SIRT3‐dependent actions of berberine. While many therapeutic effects are strongly supported by genetic loss‑of‑function or rescue experiments, other reported interactions rely mainly on association or downstream signaling and warrant further experimental validation. This classification enhances mechanistic clarity and provides a more rigorous framework for interpreting the context‐dependent roles of SIRT3 in mediating the diverse pharmacological and toxicological actions of berberine.

## 7 Conclusion

Collectively, current evidence suggests that SIRT3 may represents a context‐responsive mediator of berberine's metabolic and cytoprotective actions rather than a direct molecular target with uniform directional modulation. The bidirectional regulation of SIRT3 observed across tissues likely reflects adaptive mitochondrial remodeling shaped by metabolic state, isoform processing, post‐translational regulation, dosage, and metabolite composition. While preclinical data support a possible important role for SIRT3 in mediating several beneficial effects of berberine, translational certainty remains limited by pharmacokinetic constraints and insufficient human validation. Future studies integrating exposure‐guided designs, metabolite characterization, and causal genetic approaches will likely be essential to clarify the therapeutic relevance of berberine–SIRT3 modulation and to define its safe and effective clinical application.

## Author Contributions

**Dareuosh Shackebaei:** validation, visualization, writing – original draft, writing – review and editing. **Seyed Shahram Miraghaee:** investigation, validation, data curation, writing – original draft, writing – review and editing. **Faegheh Farhadi:** data curation, methodology, investigation, writing – original draft, writing – review and editing. **Abolfazl Akbarzadeh:** writing – review and editing, visualization. **Fatemeh Yarmohammadi:** conceptualization, writing – review and editing, supervision.

## Funding

The authors have nothing to report.

## Conflicts of Interest

The authors declare no conflicts of interest.

## Data Availability

The data that support the findings of this study are available from the corresponding author upon reasonable request.
